# A socio-ecological analysis of risk, protective and promotive factors for the mental health of Burundian refugee children living in refugee camps

**DOI:** 10.1007/s00787-020-01649-7

**Published:** 2020-09-21

**Authors:** Florian Scharpf, Getrude Mkinga, Faustine Bwire Masath, Tobias Hecker

**Affiliations:** 1 Department of Psychology, Bielefeld University, P. O. Box 100131, 33501 Bielefeld, Germany; 2 Vivo International, Konstanz, Germany; 3 Department of Educational Psychology and Curriculum Studies, Dar es Salaam University College of Education, Dar es Salaam, Tanzania; 4 Department of Psychology, University of Zurich, Zurich, Switzerland

**Keywords:** Refugee children, Ecological, Risk factors, Mental health, Resilience, Post-traumatic stress

## Abstract

Children and adolescents’ mental health risk and resilience arise from a complex interplay of factors on several socio-ecological levels. However, little is known about the factors that shape the mental health of refugee youth living in refugee camps close to ongoing conflict. We conducted a cross-sectional study with a representative sample of 217 Burundian refugee children aged 7–15 and their mothers residing in refugee camps in Tanzania to investigate associations between risk, protective and promotive factors from various ecological levels (individual, microsystem, exosystem), and children’s post-traumatic stress disorder (PTSD) symptoms, internalizing and externalizing problems, and prosocial behavior. Data were collected using structured clinical interviews and analyzed using multiple regression models. Exposure to violence across all contexts and engagement coping were risk factors for PTSD symptoms and internalizing problems, while only violence by mothers seemed to increase children’s vulnerability for externalizing problems. A differential impact of violence exposures on prosocial behavior was observed. Higher-quality friendships appeared to protect youth from PTSD symptoms and externalizing problems, while they also promoted children’s prosocial behavior, just as mothers’ social support networks. Prevention and intervention approaches should integrate risk, protective and promotive factors for refugee youth’s mental health across multiple ecological contexts and take into account context-specific and adaptive responses to war and displacement.

## Introduction

Refugee children and adolescents are at an increased risk of developing mental health problems due to their exposure to violence before their flight, potentially traumatizing experiences during their journey and daily stressors after their arrival in the host country [1, 2]. Accordingly, high prevalence rates of trauma-related psychopathology, such as post-traumatic stress disorder (PTSD), internalizing problems (e.g., depression and anxiety), and externalizing problems (e.g., aggressive and antisocial behavior), have been found among refugee youth [3, 4]. A better understanding of the factors which increase and alleviate refugee children’s vulnerability for mental health problems is urgently needed to develop targeted prevention and intervention strategies [2].

Healthy child development has been widely depicted using ecological systems theory [5], which describes development as interactions between children and their social environment across five nested contexts: ontogenetic level (individual attributes), microsystem (family and peers), mesosystem (interactions between microsystems), exosystem (wider community context) and macrosystem (society and culture). Such a socioecological framework has been recently applied to conceptualize the factors that contribute to the mental health and well-being of refugee and other conflict-affected children [2, 6, 7]. On the one hand, the model’s broad view takes into account the shattering direct and indirect impact of war and displacement on all aspects of children’s worlds. On the other hand, the model enriches this focus on risk factors with a resilience perspective, which considers those ecological factors that may be related to reduced mental health problems, i.e. protective factors, and to increased adjustment, i.e. promotive factors [8]. From a socio-ecological perspective, these factors are expected to have both direct and indirect effects on children’s mental health through their interrelations with factors from the same and other ecological levels [6].

On the ontogenetic level, a higher exposure to war-related traumatic events is a powerful risk factor for refugee children’s mental health across settings [2, 9]. Cumulative pre-migration trauma predicted higher levels of PTSD symptoms, depression, and anxiety in longitudinal studies with refugee minors in Norway [10] and Belgium [11]. Although the way refugee children cope with their adverse experiences is likely crucial for their mental health, there has been little research on the role of coping strategies [7]. In the coping literature, engagement coping strategies, including problem-focused coping, support seeking, emotion regulation and cognitive restructuring, have been generally associated with positive mental health outcomes [12]. In a study with war-affected Bosnian adolescents [13], a greater use of engagement strategies was associated with lower levels of PTSD symptoms. Syrian children who coped by acquiring social support and trying to reframe events also reported fewer PTSD symptoms [14]. However, greater use of problem-focused coping strategies was related to PTSD in Bosnian adolescents who were not entitled to asylum [15]. Consequently, evidence on the nature of engagement coping in terms of risk or resilience for refugee youth’s mental health is still inconclusive and limited to PTSD as an outcome.

Within the microsystem, refugee parents’ well-being and their parenting behaviors are likely to be important determinants of their children’ adjustment [9, 16]. Research in post-conflict settings has established a link between war trauma, family violence against children, and children’s mental health problems [17]. Recent studies found that refugee parents’ mental health problems stemming from exposure to war trauma and post-migration stressors were associated with greater levels of harsh parenting, which were in turn related to children’s emotional and behavioral problems [18, 19]. Adolescents living in refugee camps in Uganda and Rwanda who reported higher exposure to physical, verbal, and sexual abuse also reported higher levels of anxiety and depression symptoms [20]. High-quality friendships, in contrast, have been shown to be an important protective factor for the mental health of children and adolescents facing adversity [21, 22]. Moreover, higher-quality relationships with peers have been linked to higher levels of adolescents’ empathy [23] and prosocial behavior [24, 25]. For refugee children, higher self-reported support from friends was related to lower levels of mental health problems and better adjustment in a systematic review [9]. Higher levels of support by peers also decreased the association between stressful life events and anxiety symptoms in a more recent study with unaccompanied minors in Germany [26]. However, the association of refugee children’s friendship quality with their mental health has not been investigated.

Focusing on the exosystem, research with non-refugee populations suggests a strong link between increased levels of violence within the wider community and youth’s mental health problems, particularly PTSD symptoms and externalizing problems [27]. Higher exposure to community violence in the United States predicted higher levels of PTSD symptoms and risk behaviors as well as worse academic outcomes in Khmer refugee adolescents [28]. However, no study has investigated the association between refugee children’s current exposure to community violence and their mental health. Given the dynamic interaction between the different socio-ecological contexts, refugee parents may draw on social resources within the community that also indirectly benefit the individual child. For instance, wider and higher-quality parental social support networks have been linked to better mental health outcomes in children and adolescents [29]. Refugee adolescents exhibited lower levels of psychopathology when they reported having families with wide kin contacts and mothers who often received visitors at home [30]. The association between parents’ social networks and child mental health is likely to be mediated by the positive impact of emotional and instrumental support on parental well-being and family functioning [31]. There is also evidence suggesting that mothers’ social network quality predicts adolescents’ own prosocial behavior through mechanisms of social learning [32].

Although 80% of refugees flee to neighboring low- and middle-income countries where they usually settle in refugee camps (UNHCR, 2019), current evidence on risk and protective factors for refugee youth’s mental health is largely based on studies conducted in high-income countries, such as Europe, North America, and Australia [2, 9]. This means that the lived realities of the majority of refugee children are not adequately represented by the current state of research. While refugee children in high-income countries may have to cope primarily with adjustment to a different host culture and complex asylum processes, those resettling in camps in low-income settings often suffer from material hardships and face ongoing threats to their security [2]. Among the studies that investigated risk and protective factors associated with the mental health of refugee youth in camps, most focused on individual-level factors and few considered the family and community context [4]. Another shortcoming of available studies is the unilateral focus on negative mental health outcomes. To understand children’s resilience in the face of severe adversity, it is also important to look at positive aspects of mental health and to identify factors which promote these outcomes [8].

Applying a socio-ecological framework, this study aimed to investigate risk, protective, and promotive factors for the mental health of Burundian refugee children and adolescents currently living in refugee camps. In 2015, opposition against the Burundian president’s illegitimate third term led members of the ruling party to commit violence and atrocities towards perceived opponents, including abductions, extrajudicial killings, and torture, and caused more than three hundred thousand Burundians to flee to neighbouring countries [33]. Based on available evidence, we expected that exposure to violence on the different ecological levels (war exposure, violence by mothers, community violence) would be associated with higher levels of mental health problems and lower levels of prosocial behavior. Due to the inconclusive findings, we did not have a priori hypotheses regarding the association between engagement coping and mental health outcomes. Based on previous findings with refugee and non-refugee youth, we expected that higher-quality relationships with friends as well as higher quality maternal social support networks would be associated with lower levels of mental health problems and higher levels of prosocial behavior.

## Methods

### Sampling and participants

The study was conducted between January and May 2018 in three large refugee camps (Nduta, Nyarugusu, and Mtendeli) in Western Tanzania. Recruitment was conducted using a combined systematic and random sampling approach to identify family triads consisting of the mother, the father, and the oldest child between 7 and 15 years, i.e. of primary school age (for details see [34]). For the current study, we focused on children and their mothers because women are primarily in charge of children’s socialization in Burundian culture [35]. The final study sample consisted of 217 children (47% female) and their mothers. As 85.6% (*n* = 186) of the primary female caregivers were children’s biological mothers, we refer to them as mothers. Other types of caregiver were relatives, such as aunts or sisters (5.6%, *n* = 12), step mothers (2.8%, *n* = 6), and foster mothers (6%, *n* = 13). The majority of the families (65.9%, *n* = 143) arrived at the camps in 2015 after the outbreak of political violence in Burundi. Most mothers (79.7%, *n* = 173) identified the political conflicts as the main reason for their flight. Table 1 shows other sociodemographic characteristics of the children and mothers.

**Table 1:** Sociodemographic characteristics of children and mothers

	Children (*n* = 217)	Mothers (*n* = 217)
Age in years, *M* (SD, range)	12.16 (2.03, 7–15)	34.74 (8.52, 19–74)
Education, % (*n*)		
No schooling	8.3 (18)	35.0 (76)
Class 1–3 (primary)	49.3 (97)	22.6 (49)
Class 4–6	39.6 (86)	30.8 (67)
Some secondary	2.8 (6)	10.7 (23)
Completed secondary^a^		0.9 (2)
Ethnicity		
Hutu	82.5 (179)	
Tutsi	14.4 (31)	
Other	3.1 (7)	
Household size, *M* (SD, range)	7.3 (1.98, 3–14)	
Household income *p*. month (USD), % (*n*)		
No income	36.4 (79)	
Up to 20	54.9 (119)	
More than 20	8.7 (19)	

^a^This was not applicable to children

### Procedure

Data collection took place on the compounds of collaborating NGOs within the camps. Upon arrival, families were provided information regarding the purpose and the procedure of the study. Then, each family member gave their informed consent by signing with their names or fingerprints. Parents gave their consent on behalf of children under the age of 11. All but two families were willing to participate in the study. The research team, consisting of Tanzanian master-level psychologists and trained research assistants from the refugee community residing in the camps, conducted structured clinical interviews individually with each parent and child in Swahili, the lingua franca in Tanzania and the refugee camps, or in Kirundi, the native language of Burundians. Trained interpreters were always present in case a participant was not proficient in Swahili (for details on the procedure see [34]). After the interviews, families received a material compensation of 8 US Dollars. The study was approved by the Ethics Commission of the University of Zurich and the Tanzanian National Institute for Medical Research. All necessary research permits were obtained from the Tanzanian Commission for Science and Technology (COSTECH) and the Tanzanian Ministry of Home Affairs.

### Measures

The interview guides for mothers and children consisted of individual questionnaires that were either already available in Swahili or were translated from English to Swahili for this study using blind-back translation [36]. Prior to data collection, we conducted focus group discussions with the Burundian research assistants to qualitatively evaluate the appropriateness of the instruments and relevant mental health concepts in Burundian culture. We also conducted a pilot assessment with eight families, which allowed us to make necessary adaptations and supported the appropriateness of the instruments for the study context.

#### Sociodemographic information

Children and mothers were asked about their age and educational level. Mothers also answered questions about their ethnicity, date of arrival at the camp, household characteristics (size, average income per month) and the reasons for their flight.

#### Post-traumatic stress symptoms

The University of California at Los Angeles Child/Adolescent PTSD Reaction Index for DSM-5 (PTSD-RI-5) [37] was used to assess children’s PTSD symptoms within the past month according to the criteria of the Diagnostic and Statistical Manual of Mental Disorders (DSM) 5th Edition [38]. The 31 items are scored on a 5-point Likert scale ranging from 0 (none of the time) to 4 (most of the time) and are summed up to a total score of PTSD symptom severity ranging from 0 to 124. The PTSD-RI-5 has been used in various cultural settings and has shown good psychometric properties [39]. It has also been applied successfully in studies with refugee youth [40]. Internal consistency of the total score was high in our sample, with Cronbach’s *α* = 0.90.

#### Internalizing and externalizing problems and prosocial behaviour

The self-report version of the Strength and Difficulties Questionnaire (SDQ) is a widely used measure to screen for emotional and behavioural problems in children and adolescents with good psychometric properties [41]. The 25 items are rated as not true (0), somewhat true (1), or certainly true (2). All items are divided between five subscales of five items each: emotional problems, peer problems, hyperactivity/inattention, conduct problems, and prosocial behavior. The subscales emotional problems and peer problems can be combined to create an internalizing problem score (range 0–20), while the sum of the subscales hyperactivity/inattention and conduct problems yields an externalizing problems score (range 0–20). The four subscales combined provide an index of total difficulties (range 0–40). The SDQ has been extensively used in Sub-Saharan Africa [42] and in refugee settings [16, 20]. Cronbach’s alpha of the total difficulties score was 0.65 in our sample, which is comparable to other studies [16, 43]. The rather low internal consistency can be attributed to the heterogeneity of the total score consisting of both internalizing and externalizing symptoms across four subscales.

#### War-related traumatic events

22 items derived from a checklist by Neuner et al. [44] were used to assess children’s exposure to war-related traumatic events (e. g. physical injury, sexual assault, dangerous flight).

#### Engagement coping

We used an adapted version of the Kidcope [45] that had been developed based on qualitative research with war-affected Congolese adolescents [46] to assess the frequency of children’s use of engagement coping strategies. It covered five engagement strategies (cognitive restructuring, social support, problem solving, emotion regulation, praying) and six disengagement strategies (withdrawal, distraction, wishful thinking, self-criticism, resignation, blaming others) with 18 items. Similar to previous work [46], we asked children first to identify one stressful situation within the past week, for example in their family, with friends, or at school, and then to indicate whether they had used a specific strategy to cope with that situation on a binary yes (1)/no (0) scale. Here, we were only interested in the five engagement strategies measured by seven items (range 0–7).

#### Violence by mothers

We used the Parent–Child Conflict Tactic Scales (CTSPC) [47] to assess the mother’s use of physical and emotional violence towards the participating child. Children reported the frequency of specific abusive acts within the past year over 18 items on a 7-point Likert scale ranging from 0 = never to 25 =  > 20 times. Summing up all items yielded a total score of maternal violence within the past year with a potential range from 0 to 450. The CTSPC self-report version has shown good psychometric properties [48] and has been used successfully in East Africa [49]. Internal consistency of the total violence score was good (*α* = 0.80).

#### Quality of relationships with friends

A shortened version [50] of the People in My Life Questionnaire (PIML) [51] was used to assess the quality of children’s relationships with their friends. The PIML was originally designed to measure children’s internal representations of their relationship with parents, friends and teachers. The 15 items referring to friends are rated on a 4-point scale from 0 (almost never or never true) to 3 (almost always or always true) and cover the subscales trust, communication and alienation [52]. The PIML has demonstrated good psychometric properties [52, 53]. Cronbach’s Alpha of the total score ranging from 0 to 45 was good (*α* = 0.82).

#### Community violence

Mothers’ report of experienced and witnessed violence committed by community members within the past month served as a proxy for children’s current exposure to community violence. The nine items answered on a binary yes (1)/no (0) scale covered different violence-related events, e.g., physical and sexual assault, and were derived from a checklist used in a study with Congolese refugees in a Ugandan refugee camp [54].

#### Maternal social support networks

Five purpose-built items assessed how often mothers had met with others in a public place, received visitors at home, visited other people and talked about problems with friends, family members or other persons with similar traumas within the past month. The items were rated on a 5-point scale from never (0) to very often (4) and summed up to receive a total score ranging from 0 to 20. Internal consistency was acceptable (*α* = 0.71).

### Data analysis

The data were analysed using the Statistical Package for the Social Sciences (SPSS) version 25.0. There were no missing values. To investigate the associations between presumed risk, protective and promotive socio-ecological factors, and the outcomes PTSD, internalizing problems, externalizing problems, and prosocial behaviour, we conducted four multiple linear regression models, one for each outcome. The predictors on the different ecological levels—exposure to war-related trauma and engagement coping on the ontogenetic level, violence by mothers and friendship quality in the microsystem, community violence and mothers’ social network in the exosystem—were entered at once in the regression models.

We retained univariate outliers in the analyses to also consider naturally occurring extreme values in children’s experiences and mental health. Multivariate outliers were removed from regression analyses. The regression models fulfilled all other necessary quality criteria for linear regression analysis. Residuals did not deviate significantly from normality, linearity, or homoscedasticity. The maximum variance inflation factor for the regression models did not exceed 1.26. Hence, we did not need to take multicollinearity into account. Children’s age and gender were added as covariates in the regression models due to their potential impact on both outcome variables and predictor variables [16]. Our metric for a small effect size was *f*^2^ ≥ 0.02, for a medium effect *f*^2^ ≥ 0.15, and for a large effect *f*^2^ ≥ 0.35 [55]. All analyses used a two-tailed *α* = 0.05.

## Results

Descriptive statistics and bivariate correlations are displayed in Tables 2 and 3. Table 4 shows the results of the multiple regression analyses. The model predicting PTSD symptoms explained 41% of the variability of PTSD symptoms [adj. *R*^2^= 0.41, *F*(8, 198) = 18.83, *p* < 0.001, *f*^2^ = 0.69]. Exposure to war-related traumatic events, violence by mothers and community violence as well as engagement coping strategies were significantly positively associated with PTSD symptoms, whereas friendship quality was significantly negatively related to PTSD symptoms. The model predicting internalizing problems explained 12% of the variability of internalizing problems [adj. *R*^2^ = 0.12, *F*(8, 194) = 4.46, *p* < 0.001, *f*^2^ = 0.14]. Exposure to war-related traumatic events, violence by mothers and community violence as well as engagement coping strategies were significantly positively associated with internalizing problems. The model predicting externalizing problems explained 12% of the variability of externalizing problems [adj. *R*^2^ = 0.12, *F*(8, 191) = 4.29, *p* < 0.001, *f*^2^ = 0.14]. Exposure to violence by mothers was significantly positively associated and friendship quality was significantly negatively associated with externalizing problems. Finally, the model predicting prosocial behavior accounted for 19% of the variability in prosocial behavior [adj. *R*^2^ = 0.19, *F*(8, 200) = 7.20, *p* < 0.001, *f*^2^ = 0.23]. Exposure to war-related and community violence, friendship quality and the quality of mothers’ social networks were significantly positively related to prosocial behavior, whereas violence by mothers was significantly negatively related to prosocial behavior.

**Table 2:** Descriptive statistics of study variables

Study variables, *M* (SD, min–max)	
Posttraumatic stress symptoms (UCLA-RI-5)	14.98 (11.31, 0–49)
Internalizing problems (SDQ)	6.36 (3.24, 0–16)
Externalizing problem (SDQ)	4.40 (2.93, 0–15)
Prosocial behavior (SDQ)	8.26 (1.57, 3–10)
War-related traumatic events	3.77 (3.68, 0–16)
Engagement coping (Kidcope)	3.87 (1.90, 0–7)
Violence by mothers (CTSPC)	42.91 (42.28, 0–214)
Friendship quality (PIML)	31.43 (8.02, 5–45)
Community violence	2.67 (2.46, 0–9)
Maternal social support network	9.47 (5.00, 0–20)

*UCLA-RI-5* University of California at Los Angeles Child/Adolescent PTSD Reaction Index for DSM-5, *SDQ* Strengths and Difficulties Questionnaire, *CPCTS* Parent–Child Conflict Tactic Scales, *PIML* People in My Life Questionnaire

**Table 3:** Bivariate correlations between study variables

Variable	1	2	3	4	5	6	7	8	9	10	11
Age	–										
PTSD symptoms (UCLA-RI-5)	0.129	–									
Internalizing problems (SDQ)	0.069	0.345***	–								
Externalizing problems (SDQ)	− 0.052	0.202**	0.314***	–							
Prosocial behavior (SDQ)	− 0.092	0.054	0.009	− 0.202**	–						
War-related trauma	0.154*	0.535***	0.255***	0.083	0.160*	–					
Engagement coping (Kidcope)	0.142*	0.189**	0.161*	− 0.073	0.054	0.176**	–				
Violence by mothers (CTSPC)	0.036	0.233**	0.079	0.176**	− 0.118	0.173*	− 0.060	–			
Friendship quality (PIML)	− 0.169*	− 0.142*	0.028	− 0.180**	0.256***	− 0.084	0.305***	− 0.186**	–		
Community violence	− 0.120	0.183**	0.131	0.045	0.073	0.139*	− 0.074	0.075	− 0.113	–	
Mothers’ network	0.006	− 0.006	− 0.040	0.067	0.225**	− 0.056	− 0.029	0.042	0.076	− 0.224**	–

*UCLA-RI-5* University of California at Los Angeles Child/Adolescent PTSD Reaction Index for DSM-5, *SDQ* Strengths and Difficulties Questionnaire, *CPCTS* Parent–Child Conflict Tactic Scales, *PIML* People in My Life Questionnaire

***p < 0.001, **p < 0.01, *p < 0.05

**Table 4:** Results of multiple regression analyses

	PTSD symptoms^a^ (*n* = 207)				Internalizing problems^b^ (*n* = 203)				Externalizing problems^c^ (*n* = 200)				Prosocial behavior^d^ (*n* = 209)			
	*B*	*β*	*t*	*p*	*B*	*β*	*t*	*p*	*B*	*β*	*t*	*p*	*B*	*β*	*t*	*p*
Sociodemographic variables																
Age	0.02	0.003	0.06	0.95	− 0.02	− 0.01	− 0.17	0.86	− 0.12	− 0.10	− 1.36	0.18	− 0.05	− 0.07	− 1.11	0.38
Gender (male = 0, female = 1)	**− 2.50**	**− 0.12**	**− 2.16**	**0.03**	− 0.59	− 0.11	− 1.57	0.12	0.59	0.12	1.73	0.09	− **0.36**	− **0.13**	− **1.98**	**0.05**
Individual																
War-related trauma	**1.50**	**0.50**	**8.88**	** < 0.001**	**0.13**	**0.20**	**2.23**	**0.03**	0.06	0.09	1.23	0.22	**0.10**	**0.26**	**3.84**	** < 0.001**
Engagement coping	**0.81**	**0.15**	**2.48**	**0.01**	**0.22**	**0.15**	**2.05**	**0.04**	− 0.08	− 0.06	− 0.85	0.40	− 0.04	− 0.05	− 0.66	0.51
Microsystem																
Violence by mothers	**0.05**	**0.19**	**3.39**	**0.001**	**0.01**	**0.17**	**2.54**	**0.01**	**0.02**	**0.23**	**3.37**	**0.001**	− **0.01**	− **0.16**	− **2.50**	**0.01**
Friendship quality	− **0.16**	− **0.12**	− **2.01**	**0.05**	0.03	0.10	1.28	0.20	− **0.07**	− **0.20**	− **2.81**	**0.005**	**0.04**	**0.24**	**3.17**	**0.002**
Exosystem																
Community violence	**0.55**	**0.13**	**2.25**	**0.03**	**0.25**	**0.22**	**3.09**	**0.002**	0.03	0.02	0.34	0.73	**0.08**	**0.13**	**2.01**	**0.05**
Maternal social network	0.09	0.04	0.77	0.45	0.04	0.07	1.00	0.32	0.07	0.08	0.13	0.06	**0.09**	**0.29**	**4.59**	** < 0.001**

^a^Adj. *R*^2^ = 0.41, *F*(8, 198) = 18.83, *p* < 0.001, *f*^2^ = 0.69

^b^Adj. *R*^2^ = 0.12, *F*(8, 194) = 4.46, *p* < 0.001, *f*^2^ = 0.14

^c^Adj. *R* ^2^ = 0.12, *F*(8, 191) = 4.29, *p* < 0.001, *f*^2^ = 0.14

^d^Adj. *R*^2^ = 0.19, *F*(8, 200) = 7.20, *p* < 0.001, *f*^2^ = 0.23

## Discussion

This study investigated risk, protective, and promotive factors across multiple socio-ecological levels (individual level, microsystem, exosystem) for negative (PTSD symptoms, internalizing and externalizing problems) and positive (prosocial behavior) mental health outcomes in Burundian refugee youth living in camps close to ongoing conflict. As expected, higher exposure to violence related to war, within the family, and in the community was associated with higher levels of PTSD symptoms and internalizing problems. This is consistent with a large body of evidence documenting the detrimental mental health impact of traumatic experiences [2, 56], child maltreatment [20, 40, 57] and community violence [27, 28] for refugee and non-refugee children and adolescents across various socioeconomic and cultural settings. Studies conducted within a socio-ecological framework have shown how violence on more distal ecological levels, e.g., structural and community violence, adversely affects children’s adjustment both directly and indirectly by increasing violence within the more proximal family context [58–60].

The unexpected findings that higher exposure to war-related and community violence was not associated with externalizing problems and with higher levels of prosocial behavior are noteworthy. This contradicts empirical findings and theoretical conceptualizations of how organized and community violence are associated with aggressive and antisocial tendencies and undermine prosocial behavior [54, 61–65]. However, preliminary evidence suggests that victimization and war trauma may also favor prosocial behaviors and orientations through positive experiences in the midst of adversity, e.g., receiving and giving care and help or learning from prosocial role models, thereby reflecting resilience [66]. A more prosocial attitude may be adaptive in times of violent intergroup conflict as cooperative group members may be more likely to be rewarded and non-cooperative members to be punished [67]. The finding fits with the observed lack of an association between both war-related and community violence with externalizing problems, which also include antisocial behaviors and may, thus, be considered the converse of prosocial behavior. In contrast, increased violence by mothers was associated with increased externalizing problems and reduced prosocial behavior, suggesting the importance of social learning through parental role models for both antisocial and prosocial behavior [62, 68]. The pattern of associations between violence on different ecological contexts, externalizing, and prosocial behaviors is in line with a recent study with unaccompanied refugee minors, which found family violence, but not organized violence, to be associated with self-reported aggression [69].

A more frequent use of engagement coping strategies in everyday situations, such as problem-focused coping and cognitive restructuring, was associated with higher levels of PTSD symptoms and internalizing problems, which is inconsistent with studies that found engagement strategies to be related to lower levels of mental health problems in refugee children and adults [13, 14, 70–72]. However, other studies found that more problem-focused coping was associated with higher levels of internalizing problems in sample of adult Syrian refugees living in Turkey close to the Syrian border [73] and with the presence of PTSD in Bosnian refugee youth waiting for the resolution of their asylum claims [15]. Our finding adds to these and suggests that engaging intensively with situations and stressors that cannot be easily changed or controlled may also be detrimental to mental health. The children in our study lived in refugee camps close to ongoing conflict and under precarious circumstances. Many of the stressful situations they had to cope with referred to experiencing or witnessing violence at home, in the community, or at school, which may have been perceived as being beyond the children’s ability to control.

In the face of severe adversity, children with closer and more supportive relationships with friends reported lower levels of PTSD symptoms and externalizing problems as well as higher levels of prosocial behavior. This is in keeping with studies with non-refugee youth showing that higher-quality peer relationships moderated the impact of complex trauma [22], family adversity [74], and peer rejection [21] on youth’s adjustment. The finding is also consistent with previous evidence on the protective role of peer support for refugee children and adolescents’ mental health [9, 26]. The positive association between friendship quality and prosocial behavior points to the importance of peer relationships for the development of prosocial behavior [68]. Higher-quality maternal social support networks appeared to have a similarly promotive impact on children’s prosocial behavior. This may be explained through social learning mechanisms, such as social behaviors, modeled by mothers in their interactions that are observed and imitated by children [32]. However, we did not observe the expected associations between maternal social network and children’s mental health problems that has been found in previous studies [29, 30]. It is possible that a wider and closer social network of mothers indirectly exposes youth to the traumas and problems of other community members, which may outweigh any positive indirect impact of maternal social networks for youth’s mental health. However, this hypothesis requires further testing.

The current study’s novel contributions to the evidence base on refugee children and adolescents’ mental health are threefold. First, the present study contributes to efforts to better understand and support the mental health and well-being of refugee youth living in refugee camps close to ongoing conflict, a population that represents the majority of refugee minors globally. Second, the study shows that risk, protective, and promotive factors from different levels in children’s social environment are relevant for their adjustment and, thus, strengthens the importance of a socio-ecological perspective in research and practical work with refugee children. Third, the study not only includes a range of different negative mental health outcomes, but also investigates prosocial behavior reflecting positive adjustment. However, some limitations must be noted. First, due to the cross-sectional study design causal interpretations of the observed associations should be made with caution. Reciprocity may also be plausible when interpreting the present findings. For instance, children’s externalizing problems have been shown to elicit physical discipline by parents [75], while more prosocial behavior also led to increased acceptance by peers [76]. Second, the specific cultural background and living context of our sample may limit the generalizability of our findings to refugee youth with other backgrounds or living in other settings, e.g., in high-income countries or urban community settings. However, we argue that the factors we included in our study are also relevant and salient in the social ecologies of children living in refugee camps in other areas of the world and, thus, assume a certain generalizability to these contexts, which nevertheless has to be examined in future studies. Third, although we ensured the suitability of our measures through focus group discussions and a pilot assessment, they have not been validated for our study sample. We recognize the importance of validating assessment tools for refugee youth in diverse settings [77], and hope that future research can address this shortcoming. Fourth, we assessed children’s prosocial behavior through their self-report, which may be affected by social desirability. Direct ratings by peers or parents may also be valuable to include for analysis. Relatedly, we used maternal reports as a proxy for children’s exposure to community violence which may underestimate the amount of children’s actual exposure. The assessment of community factors, such as violence or social support, from the child perspective or through more objective indicators may be useful in future studies.

Our findings may have important implications for the practical clinical work with refugee youth in camp settings. Interventions should target factors on different ecological levels, i.e. related to the individual child, family, peers and the community, and various mental health outcomes, i.e. negative and positive aspects of adjustment. Considering the strong detrimental impact of war-related trauma and ongoing violence, we advocate programs with a trauma focus. As violence by mothers broadly impairs children’s mental health, approaches should include parents and provide access to non-violent parenting strategies. The potentially protective and promotive nature of positive peer relationships may be harnessed by also considering interpersonal relationship skills as targets for intervention, incorporating psychosocial recreational elements (e.g., child-friendly spaces within the refugee camp), and by the choice of setting, for example, conducting interventions in a group format or in a school setting. Utilizing existing community spaces and incorporating lay personnel into care provision would also require fewer additional resources in camp settings that face severe resource deficits. Examples for possible interventions include trauma-focused cognitive behavioral therapy [78] and its adaption teaching recovery techniques [79], which have been tested in studies with war-affected Congolese girls [80] and unaccompanied refugee minors in Sweden [81], respectively. These programs also focus on coping with trauma and distress. Most notably, our findings suggest that it is important to take into account the context-dependent nature of certain coping styles and to carefully evaluate whether these may be adaptive or maladaptive for youth. Similarly, potential positive outcomes in the face of trauma and violence, such as an increased prosocial orientation, need to be considered by practitioners working in prevention and intervention.

In conclusion, exposure to violence across all contexts and engagement with everyday stressors appears to increase refugee youth’s risk for mental health problems, but there is also evidence for peer and community resources contributing to resilience in the midst of war and displacement. Prevention and intervention approaches aiming to improve and promote refugee children’s mental health should focus on reducing children’s exposure to violence in their social ecology and strengthen their relationships with peers.
